# Horner’s syndrome: an unusual complication of thyroidectomy: a case report

**DOI:** 10.1186/s13256-016-1072-7

**Published:** 2016-10-26

**Authors:** Sanjeewa A. Seneviratne, Dewamuni S. Kumara, Akram M. P. Drahaman

**Affiliations:** 1 Department of Surgery, University of Colombo, Colombo, Sri Lanka; 2 National Hospital of Sri Lanka, Colombo, Sri Lanka

**Keywords:** Horner’s syndrome, Thyroidectomy, Cervical sympathetic chain damage, Case report

## Abstract

**Background:**

Horner’s syndrome is a very rare complication following surgery of the thyroid gland with only a handful of cases reported in the literature. Exact pathophysiology of post-thyroidectomy Horner’s syndrome is not fully understood, and once diagnosed, management remains mostly conservative.

**Case presentation:**

A 36-year-old Sri Lankan Sinhalese woman developed unilateral partial ptosis with enophthalmos and myosis one week after total thyroidectomy for a benign multinodular goiter. A clinical diagnosis of Horner’s syndrome was made. A postoperative ultrasound scan did not show a collection or hematoma compressing the sympathetic trunk. Our patient was managed conservatively and she had a slow and an incomplete recovery at 1-year follow up.

**Conclusions:**

This case report highlights the importance of being aware of the close anatomical relationship between the thyroid gland and cervical sympathetic trunk during thyroidectomy. This would enable the surgeon to undertake measures to minimize the risk of damaging the sympathetic trunk during thyroidectomy.

## Background

Horner’s syndrome (HS) is characterized by myosis, eyelid ptosis, enophthalmos, with or without facial anhydrosis and vascular dilatation of one half of the face resulting from damaged ipsilateral cervical sympathetic chain. HS due to damaged cervical sympathetic trunk during thyroidectomy is a very rare complication with only a handful of cases reported in the literature [1, 2]. A close and highly variable anatomical relationship between the thyroid gland and cervical sympathetic trunk places the sympathetic trunk at increased risk of damage during thyroidectomy. We report a case of HS developing after thyroidectomy for an uncomplicated benign multinodular goiter where no undue difficulties were encountered during the surgery. This case is interesting in the fact that it was characterized by late onset, and incomplete recovery [2].

## Case presentation

A 36-year-old Sri Lankan Sinhalese woman underwent a total thyroidectomy for a benign, euthyroid, moderate-sized multinodular goiter with no retrosternal extension. She did not have any other medical illnesses or previous surgical procedures in the neck area. No undue difficulty or unexpected findings were encountered during the surgery. Both recurrent laryngeal nerves and superior parathyroid glands in both sides were identified and preserved with the standard technique and branches of both inferior thyroid arteries were ligated and divided close to the capsule of the gland.

The early postoperative period was uncomplicated and our patient was discharged from the ward on the first postoperative day. Six days later (a week after thyroidectomy) she was detected to have left-sided partial ptosis with enophthalmos and myosis. However, there was no anhydrosis or vascular dilatation in the ipsilateral face. She did not develop other complications of thyroidectomy including hypoparathyroidism or vocal cord palsy. An ultrasound scan of her neck failed to demonstrate a hematoma or a collection which potentially could have compressed the sympathetic trunk. Pathological analysis of the specimen showed a multinodular colloid goiter without any evidence of malignancy.

Our patient was managed conservatively and her recovery from HS was slow and incomplete. By 6 months she showed a slight improvement in ptosis and myosis, but no further improvements noted at 1-year follow up.

## Discussion

Horner’s syndrome (HS) was described first in 1853 by Bernard, and then in 1869 by Swiss ophthalmologist Johann Horner [3]. HS is characterized by myosis, eyelid ptosis, enophthalmos, with or without facial anhydrosis and vascular dilatation of one half of the face resulting from damaged ipsilateral cervical sympathetic chain.

Although there are frequent reports of HS caused by compression of the cervical sympathetic chain by a large benign goiter or infiltration by a malignant goiter, HS as a complication of thyroidectomy as described in this case report is a very rare entity, with less than 30 cases so far reported in the literature [2]. Furthermore, the majority of such reported cases are following surgery for malignant thyroid glands combined with lymph node dissection or following complicated thyroid surgery.

Kappeler was the first to describe HS as a complication of thyroid surgery in 1865 and the first reported case of HS following thyroidectomy was by Kaelin in 1915 [4]. While the association between traditional thyroidectomy and HS is well known, recent reports suggest that similar risks of damage to sympathetic chain are associated with minimal access surgery as well. For instance, Harding *et al*. has recently reported a case of HS following minimally invasive parathyroidectomy [2], while Meng and colleagues have reported HS following minimally invasive video-assisted thyroidectomy [5].

**Fig. 1:**
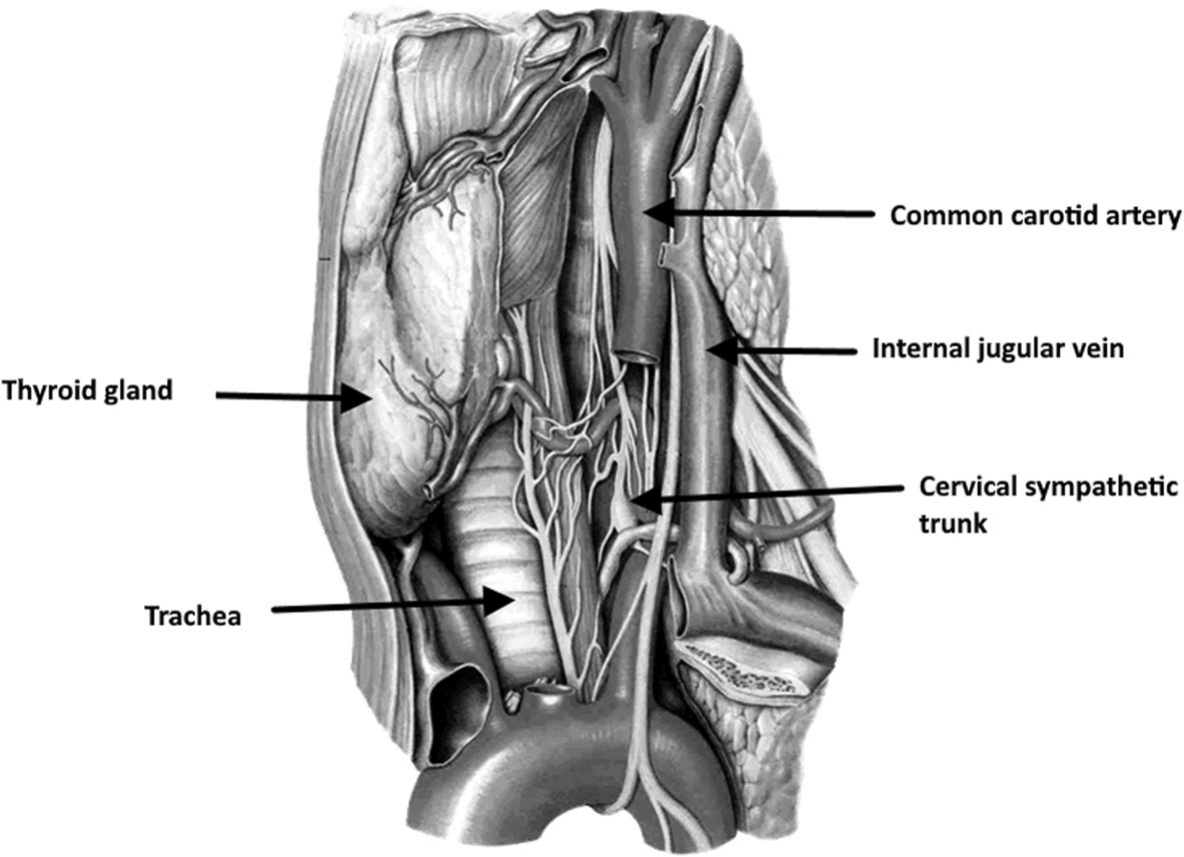
An illustration demonstrating the cervical sympathetic pathway and its anatomical relationship to the thyroid gland

There are several theories as to the possible reasons for HS after thyroidectomy. These include stretching of the cervical sympathetic chain during lateral retraction, postoperative hematoma compressing the cervical sympathetic chain, ischemia-induced neural damage caused by a lateral ligature on the inferior thyroid artery trunk and damage to the communication between the cervical sympathetic chain and the recurrent laryngeal nerve during its identification [2]. The middle cervical ganglion and the sympathetic trunk lie close and in a variable relationship, lying either in front or behind the inferior thyroid artery as the artery arches medially from the thyrocervical trunk (Fig. 1). This close, and more importantly variable, anatomical relationship makes the sympathetic trunk and the middle cervical ganglion highly susceptible during thyroidectomy. Solomon *et al.* have postulated a possible vascular supply to the sympathetic trunk originating from the inferior thyroid artery or its branches and ligation of these vessels may lead to devascularization and ischemic injury to the sympathetic chain leading to HS [6].

In 1915 de Quervain described that the stretching of the sympathetic chain during lateral retraction of the carotid sheath to assist the exposure of the lateral aspect of the gland and its associated structures might be sufficient to cause trauma to the chain with a subsequent neuropraxic-type injury [7]. In a recently published case series Harding *et al*. have shown that the risk of HS increases with more extensive surgery or complicated thyroid surgery, for instance in malignant thyroid disease with level III lymph node dissection and with surgery for large goiters with retrosternal extension [2].

There is no uniformity in the onset of HS after thyroidectomy and the literature shows most cases having the onset on 2^nd^ to 4^th^ postoperative day reflecting the possibility of different etiologies [1]. The case described here is unique due to the fact that onset of HS was very late at 1 week following thyroidectomy. This is later than any reported cases so far in the literature and may be due to delayed ischemic injury to the sympathetic trunk or due to so far unrecognized etiology.

Most reported cases of post-thyroidectomy HS patients did not have symptoms related to the vascular system that includes ipsilateral facial anhydrosis and cutaneous vascular dilatation [1, 2]. The exact reason for this is unclear and the case described also had no anhydrosis and vascular dilatation.

We suspect that HS in the patient reported here was produced by ischemic damage combined with possible local trauma to the sympathetic chain during retraction of the carotid sheath. Delayed onset, partial nature of damage to the sympathetic trunk and incomplete recovery support this theory. However, a postoperative hematoma compressing the sympathetic trunk is another possibility, although such an abnormality was not seen with the postoperative ultrasound scan.

## Conclusions

This case report highlights a rare but important complication of a very common surgery undertaken by general as well as endocrine and otorhinolaryngology surgeons. Although it is rare, the operating surgeon should appreciate the importance of avoiding injury to this at-risk structure since it leads to a significant cosmetic disfigurement, which could be permanent in a significant minority.

## Abbreviations

HS: Horner’s syndrome
